# Symmetry-enforced topological Dirac semimetal for giant spin–orbit torque with ultralow power dissipation

**DOI:** 10.1093/nsr/nwag077

**Published:** 2026-02-04

**Authors:** Xuan Zheng, Siyang Peng, Xuejiao Chen, Bin Lao, Yamin Han, Liguang Gong, Tao Tang, Keyi Wu, Yan Sun, Peitao Liu, Xianfeng Hao, Youguo Shi, Nicholas C Plumb, Ming Shi, Tao Wu, Shouzhong Peng, Xing-Qiu Chen, Zhicheng Zhong, Milan Radovic, Run-Wei Li, Zhiming Wang

**Affiliations:** Zhejiang Province Key Laboratory of Magnetic Materials and Applications, Ningbo Institute of Materials Technology and Engineering, Chinese Academy of Sciences, Ningbo 315201, China; New Materials Institute, University of Nottingham Ningbo China, Ningbo 315100, China; Zhejiang Province Key Laboratory of Magnetic Materials and Applications, Ningbo Institute of Materials Technology and Engineering, Chinese Academy of Sciences, Ningbo 315201, China; School of Artificial Intelligence and Data Science, University of Science and Technology of China, Suzhou 215123, China; School of Photoelectric Engineering, Changzhou Institute of Technology, Changzhou 213032, China; Zhejiang Province Key Laboratory of Magnetic Materials and Applications, Ningbo Institute of Materials Technology and Engineering, Chinese Academy of Sciences, Ningbo 315201, China; Zhejiang Province Key Laboratory of Magnetic Materials and Applications, Ningbo Institute of Materials Technology and Engineering, Chinese Academy of Sciences, Ningbo 315201, China; Zhejiang Province Key Laboratory of Magnetic Materials and Applications, Ningbo Institute of Materials Technology and Engineering, Chinese Academy of Sciences, Ningbo 315201, China; Zhejiang Province Key Laboratory of Magnetic Materials and Applications, Ningbo Institute of Materials Technology and Engineering, Chinese Academy of Sciences, Ningbo 315201, China; Zhejiang Province Key Laboratory of Magnetic Materials and Applications, Ningbo Institute of Materials Technology and Engineering, Chinese Academy of Sciences, Ningbo 315201, China; Shenyang National Laboratory for Materials Science, Institute of Metal Research, Chinese Academy of Sciences, Shenyang 110016, China; Shenyang National Laboratory for Materials Science, Institute of Metal Research, Chinese Academy of Sciences, Shenyang 110016, China; Key Laboratory of Applied Chemistry, College of Environmental and Chemical Engineering, Yanshan University, Qinhuangdao 066004, China; Institute of Physics, Chinese Academy of Sciences, Beijing 100190, China; PSI Center for Photon Science, Paul Scherrer Institut, Villigen 5232, Switzerland; PSI Center for Photon Science, Paul Scherrer Institut, Villigen 5232, Switzerland; Center for Condensed Matter and School of Physics, Zhejiang University, Hangzhou 310058, China; New Materials Institute, University of Nottingham Ningbo China, Ningbo 315100, China; Fert Beijing Research Institute, National Key Lab of Spintronics, School of Integrated Circuit Science and Engineering, Beihang University, Beijing 100191, China; Shenyang National Laboratory for Materials Science, Institute of Metal Research, Chinese Academy of Sciences, Shenyang 110016, China; School of Artificial Intelligence and Data Science, University of Science and Technology of China, Suzhou 215123, China; PSI Center for Photon Science, Paul Scherrer Institut, Villigen 5232, Switzerland; Zhejiang Province Key Laboratory of Magnetic Materials and Applications, Ningbo Institute of Materials Technology and Engineering, Chinese Academy of Sciences, Ningbo 315201, China; Ningbo Institute of Digital Twin, Eastern Institute of Technology, Ningbo 315200, China; Zhejiang Province Key Laboratory of Magnetic Materials and Applications, Ningbo Institute of Materials Technology and Engineering, Chinese Academy of Sciences, Ningbo 315201, China; Center of Materials Science and Optoelectronics Engineering, University of Chinese Academy of Sciences, Beijing 100049, China

**Keywords:** topological Dirac semimetal, spin-orbit torque, transition metal oxide, nonsymmorphic symmetry, magnetization switching

## Abstract

Current-driven spin–orbit torque (SOT) enables electrical control of magnetization for next-generation memory and logic, but reducing switching current and power consumption is still a major challenge. Topological semimetals provide an attractive platform because they combine metallic conductivity with topological states that can efficiently generate spin currents. However, while most studied systems rely on accidental band inversions, the SOT response of symmetry-enforced Dirac semimetals remains largely unexplored. Here, we demonstrate that the non-symmorphic symmetry-enforced Dirac semimetal hexagonal SrIrO_3_ exhibits record-high SOT efficiency. *In situ* angle-resolved photoemission spectroscopy on high-quality epitaxial thin films directly confirmed the topological Dirac semimetal state, revealing bulk Dirac points near the Fermi level and spin–momentum locked surface states. Leveraging these synergistic features, we achieve a very high SOT efficiency of 2.26 and a substantial spin Hall conductivity of 0.96 × 10^5^ ($\mathrm{h}$^-^ /2e) Ω^−1^ m^−1^, enabling perpendicular magnetization switching at an exceptionally low current density of 5.9 × 10^5^ A/cm^2^. Our findings establish symmetry-enforced topological semimetals as a robust materials platform for achieving superior charge–spin conversion, opening a pathway toward ultra-low-power spintronic devices.

## Teaser

Symmetry-enforced Dirac semimetal hexagonal SrIrO3 delivers record-high spinorbit torque efficiency, enabling perpendicular switching at ultralow current and pointing to ultra-low-power spintronic memory.

## INTRODUCTION

The relentless pursuit of energy-efficient spintronic devices is paramount for addressing the escalating power demands in modern electronics, particularly for data storage and processing [1–3]. Among various emerging technologies, spin–orbit torque magnetic random-access memory (SOT-MRAM) holds immense promise for non-volatile memory applications, offering potential advantages in speed and endurance [4,5]. The core mechanism, spin–orbit torque (SOT), enables efficient manipulation of magnetization [6,7]. However, a critical barrier hindering their widespread adoption and the realization of their full potential is the persistently high write currents required for magnetization switching [5,8]. This directly translates to substantial power consumption, undermining their energy efficiency advantages. Such high operational currents not only compromise device endurance and reliability due to Joule heating and electromigration but also impede efforts towards further miniaturization and high-density integration essential for future high-density memory and logic devices. Consequently, these limitations restrict the scalability required to meet the demanding performance and energy targets of future electronic systems. Therefore, there is an urgent and compelling need to discover and

engineer novel spintronic materials and mechanisms that can deliver substantially enhanced SOT efficiency and enable ultra-low power magnetization switching.

Among these, topological quantum materials (TQMs) have garnered significant attention due to their unique electronic band structures and potentially superior charge-to-spin conversion capabilities, offering a promising route to address these challenges [9–18]. Topological semimetals, including Dirac and Weyl semimetals, are particularly noteworthy within TQMs, as exotic bulk electronic states—characterized by protected band crossings and large Berry curvature—along with spin–momentum locked surface states, provide intrinsic mechanisms for generating large SOT efficiencies [12,17–27]. Central to discovering and tailoring such topological phases for spintronics is the fundamental role of crystal symmetry, which dictates the electronic band structure and protects novel topological states [28–30]. Among the diverse symmetry classes, non-symmorphic crystal symmetries, in particular, present a uniquely compelling, yet underexplored, design principle for engineering topological phases with superior spintronic functionalities. Unlike symmorphic symmetries that stabilize or protect the accidental band crossings induced by band inversion, non-symmorphic symmetries—involving fractional translations coupled with point group operations—can themselves drive and protect the emergence of topological states by enforcing band crossings at specific high-symmetry points or lines in the Brillouin zone [31–37]. Leveraging this inherent enforcement, manipulating non-symmorphic symmetry can therefore actively produce novel topological states with enhanced robustness against perturbations. Consequently, non-symmorphic symmetry-enforced topological Dirac semimetals, by virtue of these guaranteed and robust bulk Dirac cones near the Fermi level, often coexisting with distinct spin–momentum locked surface states, are theoretically poised to offer exceptional synergistic electronic features for achieving unprecedented SOT generation. Despite this profound theoretical promise stemming from their fundamental symmetry protection, the experimental realization and systematic investigation of SOT performance in these highly robust topological systems remain crucial and largely underexplored, representing a significant opportunity for advancing energy-efficient spintronics.

The transition metal oxide SrIrO_3_ offers a prime platform to explore non-symmorphic symmetry-driven topological physics and its spintronic applications. While its orthorhombic phase has garnered attention for SOT studies [38–48], the hexagonal polymorph of SrIrO_3_, characterized by face-sharing IrO_6_ octahedra, presents a distinct avenue. This hexagonal phase is theoretically predicted to host non-symmorphic symmetry-protected Dirac points near the Brillouin zone boundary [49,50]. However, the experimental verification of this topological Dirac semimetal state in hexagonal SrIrO_3_ and a systematic investigation of its associated SOT properties remain crucial yet largely unexplored tasks. Such exploration is vital for understanding how non-symmorphic symmetries can be harnessed to engineer topological states for enhanced spin–charge conversion.

Employing a multifaceted approach that combines *in situ* angle-resolved photoemission spectroscopy (ARPES), advanced transport characterization (including non-linear transport and second harmonic Hall measurements), and first-principles calculations, we unequivocally confirm the presence of both spin–momentum locked surface states and bulk 3D Dirac points near the Fermi level, firmly establishing the topological Dirac semimetal nature of hexagonal SrIrO_3_. Remarkably, this non-symmorphic topological Dirac semimetal exhibits a giant SOT efficiency reaching approximately 2.26, surpassing most reported values in topological semimetals [12,17–27]. Leveraging this exceptional efficiency, we successfully demonstrate current-driven perpendicular magnetization switching in SrIrO_3_/ferromagnet heterostructures at a low current density of 5.9 × 10^5^ A/cm^2^, underscoring its significant potential for energy-efficient spintronic applications. These findings not only advance our fundamental understanding of the intricate interplay between non-symmorphic symmetry, electronic topology and emergent spin-related phenomena but also establish hexagonal SrIrO_3_ as a highly promising material platform. More broadly, our work highlights non-symmorphic symmetry as a powerful design principle for discovering and engineering novel spintronic materials with superior charge-to-spin conversion efficiency, paving the way for the development of ultra-low-power spintronic devices.

## RESULTS

### Hexagonal SrIrO_3_: a non-symmorphic Dirac semimetal

**Figure 1.**
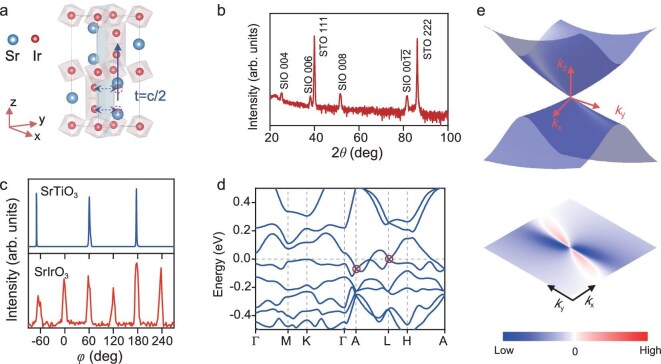
Hexagonal SrIrO_3_ as a non-symmorphic Dirac semimetal. (a) Primitive unit cell of hexagonal SrIrO_3_, composed of both corner-shared and face-shared octahedra. The non-symmorphic c-glide symmetry is demonstrated by the fact that the unit cell transforms to itself after a reflection and half-translation. (b) XRD θ–2θ scan showing the labeled peaks of the SrTiO_3_ substrate and hexagonal SrIrO_3_. (c) XRD *φ* scan of (11–2) peak of SrTiO_3_ substrate and the adjacent (155) peak of the SrIrO_3_ film, revealing the 6-fold symmetry of hexagonal SrIrO_3_. (d) Band structure calculated by DFT with Dirac points at A and L points near the Fermi energy, protected by the non-symmorphic lattice symmetry. (e) Energy dispersion and Berry curvature distribution around the Dirac point at L point near Fermi energy, revealing the Dirac point as a source of large Berry curvature.

SrIrO_3_ films with thicknesses of 27 nm were grown on SrTiO_3_(111) substrates by pulsed laser deposition (PLD). The SrTiO_3_(111) substrate exhibits 3-fold rotational symmetry (Fig. 1b), which can influence the growth and symmetry of the SrIrO_3_ film. In contrast to widely investigated orthorhombic SrIrO_3_ grown on SrTiO_3_(001) with only corner-shared oxygen octahedra, the hexagonal SrIrO_3_ grown on SrTiO_3_(111) hosts both face-shared and corner-shared oxygen octahedra (see Fig. S1). The X-ray diffraction (XRD) pattern θ–2θ and *φ* scans, shown in Fig. 1c and d, confirm that the SrIrO_3_ film adopts a hexagonal structure with 6-fold symmetry and an out-of-plane lattice constant *c* of 14.14 Å [51]. Experimental and theoretical studies suggest that the hexagonal SrIrO_3_ may prefer the *P*$\!\!\bar{\,\,\,3}$1*c* (no. 163) space group (see Supplementary data), possessing *C*_3_ rotational symmetry consistent with the substrate symmetry. Importantly, both the substrate-influenced *P*$\!\!\bar{\,\,\,3}$1*c* structure and the bulk *C*_2/*c*_ structure possess non-symmorphic symmetry, including glide symmetry and screw axis, as shown in Fig. 1a. The theoretical analysis indicates that non-symmorphic symmetry induces protected 4-fold degeneracy at the boundaries of the Brillouin zone, which is crucial for the formation of Dirac points in a class II Dirac semimetal (see Supplementary data) [31,52]. Based on density functional theory (DFT) calculations, we further confirm the existence of linear crossing Dirac points at the L and A high-symmetry points near the Fermi energy in SrIrO_3_ with the *P*$\!\!\bar{\,\,\,3}$1*c* space group (Fig. 1d). Notably, the same Dirac points are obtained at the boundary of the Brillouin zone in different space groups, consistent with previous reports in monoclinic SrIrO_3_ [49,50]. This highlights the importance of non-symmorphic symmetry in stabilizing these topological features, irrespective of the specific space group. As the Dirac points are located close to the Fermi level, the Berry curvature distribution around the Dirac point at L is of particular interest. Indeed, the calculation, shown in Fig. 1e, reveals that the Dirac point at L near the Fermi level acts as a hotspot of Berry curvature. Consequently, the large Berry curvature contribution from the Dirac point at L near the Fermi energy is expected to give rise to a significant spin Hall conductance.

### *In situ* ARPES measurements

**Figure 2.**
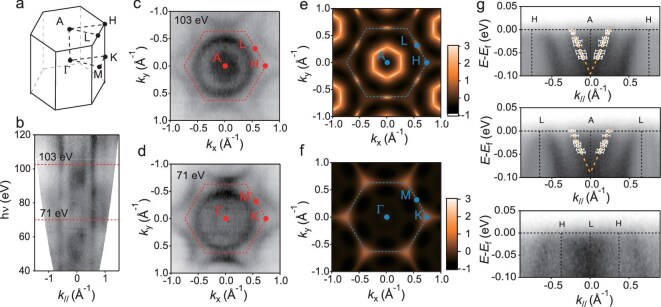
Electronic structure of hexagonal SrIrO_3_ revealed by ARPES. (a) Schematic of Brillouin zone of hexagonal SrIrO_3_. (b) Isoenergy intensity map near the Fermi energy when varying incident photon energy, showing the strong 2D character of the surface states. (c and d) Isoenergy intensity map near Fermi energy in *xy* plane at photon energy of 71 and 103 eV, respectively, corresponding to the *Γ* and A planes of the Brillouin zone. (e and f) Calculated Fermi surfaces at *Γ* and A plane. (g) Energy dispersion along L–A–L, H–A–H and H–L–H measured at a photon energy of 103 eV. The symbols show the peak fits according to the second derivative in Fig. S8a.

To directly observe the electronic structure and experimentally confirm the existence of the Dirac points predicted by theoretical calculation in hexagonal SrIrO_3_, we performed *in situ* ARPES measurements on epitaxially grown hexagonal SrIrO_3_ thin films. Figure 2c and d show the Fermi surface mappings measured at photon energies of 103 and 71 eV, which correspond to those at the A–L–H plane and Γ–M–K plane of the Brillouin zone (Fig. 2a), respectively. The Fermi surface map at the A–L–H plane (Fig. 2c) exhibits a circular electron pocket centered at the A point, in excellent agreement with the DFT calculations shown in Fig. 2e. In contrast, absence of such feature is observed at the Γ-M-K plane (Fig. 2d), also consistent with the DFT calculations shown in Fig. 2f. The energy dispersion maps along the high symmetry directions L–A–L and H–A–H (Fig. 2g) reveal an electron-like pocket at the A point with linear dispersion, corresponding to the predicted Dirac point at ~0.1 eV below the Fermi energy. The calculations also predict a Dirac point at the L point. However, the linear dispersion of this bulk Dirac cone is overshadowed by the robust 2D surface states at this location, which exhibit a parabolic dispersion, as discussed below. To further confirm the 3D nature of the Dirac point at A, we analyzed the energy dispersion around the A point across the entire Brillouin zone (see Fig. S8). Remarkably, the observation of linear dispersion along all three high-symmetry directions provides compelling evidence for the existence of a 3D Dirac point at the A point in hexagonal SrIrO_3_.

In addition to the 3D Dirac point at A, our ARPES measurements reveal the presence of surface states with a distinct 2D character, further highlighting the complexity of the topological electronic structure of hexagonal SrIrO_3_. The Fermi surface maps (Fig. 2c and d) exhibit prominent circular features that are notably absent in the calculated bulk band structure (Fig. 2e and f). By systematically analyzing the energy dispersion across different photon energies, we find that the bands corresponding to these circular features show no variation with photon energy, confirming their 2D nature (Fig. 2b). This observation strongly suggests the existence of well-defined 2D surface states in hexagonal SrIrO_3_. To further corroborate the presence of these surface states, we compare our experimental findings with the calculated surface band structure (see Supplementary data). The good agreement between the theoretical and experimental results provides additional evidence for the 2D surface states in our system. To conclude, our ARPES measurements reveal the coexistence of a 3D Dirac point at *A* and distinct 2D surface states, highlighting the complex and rich topological electronic structure of hexagonal SrIrO_3_.

### Non-linear transport measurements

**Figure 3.**
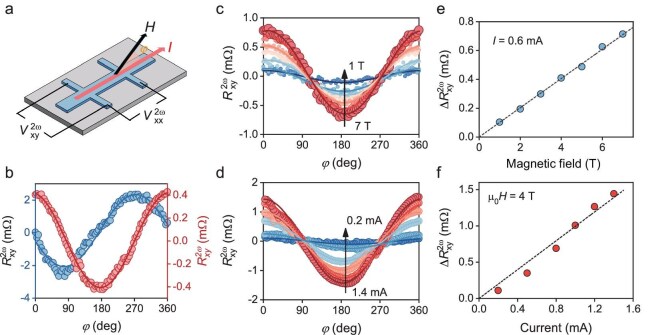
Spin–momentum locking in hexagonal SrIrO_3_ probed by non-linear transport. (a) Schematic of the Hall bar used for measurements of non-linear Hall and magnetoresistance in hexagonal SrIrO_3_ films. The angle between the magnetic field and current direction is defined as φ. (b) Angle φ dependence of non-linear Hall resistance $R_{xy}^{2\omega }$ fitted with ${\mathrm{\Delta }}R_{xy}^{2\omega }cos{\mathrm{\varphi }}$ and non-linear magnetoresistance $R_{xx}^{2\omega }$ fitted with ${\mathrm{\Delta }}R_{xx}^{2\omega }sin{\mathrm{\varphi }}$. The data were measured at a temperature of 10 K, under a magnetic field of 4 T and current of 0.6 mA. (c) Angle φ dependence of non-linear hall resistance $R_{xy}^{2\omega }$ fitted with ${\mathrm{\Delta }}R_{xy}^{2\omega }cos{\mathrm{\varphi }}$ at magnetic field varying from 7 to 1 T and current of 0.6 mA at 10 K. (d) Angle φ dependence of non-linear Hall resistance $R_{xy}^{2\omega }$ fitted with ${\mathrm{\Delta }}R_{xy}^{2\omega }cos{\mathrm{\varphi }}$ at current varying from 1.4 to 0.2 mA and magnetic field of 4 T at 10 K. (e) Magnetic field dependence of ${\mathrm{\Delta }}R_{xy}^{2\omega }$ fitted from data in (c). (f) Current dependence of ${\mathrm{\Delta }}R_{xy}^{2\omega }$ fitted from data in (d). The angle, magnetic field and current dependence of the non-linear Hall effect provide strong evidence for spin–momentum locking in the surface states of hexagonal SrIrO_3_.

Non-linear transport properties of a non-magnetic material under an external magnetic field are closely related to the electronic structure and spin texture of the surface states [53–56]. A non-linear planar Hall effect (NPHE) has been observed in orthorhombic perovskite SrIrO_3_ thin films originating from the spin–momentum locked states due to inversion symmetry broken at the surface [57]. Therefore, non-linear transport measurements can provide insight into the spin texture of the surface states observed in the ARPES measurement. Figure 3 shows non-linear transport measurements performed on a hexagonal SrIrO_3_ film. The second harmonic transverse resistance $R_{xy}^{2\omega }$ and longitudinal resistance $R_{xx}^{2\omega }$ were measured as a function of the angle ${\mathrm{\varphi }}$ between the in-plane magnetic field and the AC current direction, as depicted in Fig. 3a. The angle-dependent longitudinal and transverse resistance are fitted by ${\mathrm{\Delta }}R_{xy}^{2\omega }sin{\mathrm{\varphi }}$ and ${\mathrm{\Delta }}R_{xx}^{2\omega }cos{\mathrm{\varphi }}$, respectively. This angle dependence and π/2 phase shift of non-linear Hall resistance can be well explained by non-linear charge–spin interconversion in spin–momentum locked states [53].

Moreover, the magnetic field and current were varied to investigate how the non-linear Hall voltage depends on their magnitude. Our data show that the linear relationship of ${\mathrm{\Delta }}R_{xy}^{2\omega }$ versus magnetic field (Fig. 3c) manifests the non-linear Hall voltage proportional to *H_y_E_x_*. The slight deviation from a linear relationship of ${\mathrm{\Delta }}R_{xy}^{2\omega }$ versus current (Fig. 3d) can be attributed to thermal effects and the varying NPHE signal at different temperatures (Fig. S4). This bilinear magnetoelectric effect reflects the complex band structure and spin textures in topological surface states, as well as Rashba states [53–55]. To quantitatively compare our result with reported studies, we calculate the coefficient ${\mathrm{\chi }} = {\mathrm{\Delta }}\rho _{xy}^{2\omega }/{E}_x{H}_y$ to be 0.051 mΩ μm^2^/*VT*), which is larger than that reported in topological insulators [53]. The observed large bilinear planar Hall effect likely originates from the contribution of surface states observed by ARPES at the L and H points. Therefore, the observed non-linear Hall and magnetoresistance confirm the existence of spin–momentum locked band structure due to inversion symmetry broken at the surface.

### SOT measurements and magnetization switching

**Figure 4.**
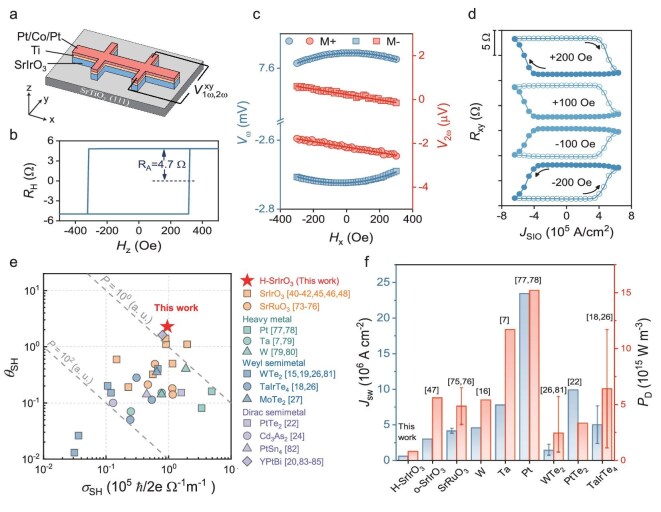
Giant SOT in hexagonal SrIrO_3_/ferromagnet heterostructures. (a) Schematic of second harmonic Hall measurement of SOT in the hexagonal SrIrO_3_/Ti/Pt/Co/Pt heterostructures at room temperature. (b) Hall measurement demonstrates perpendicular magnetic anisotropy of the ferromagnetic layer. (c) First (left axis) and second harmonic (right axis) voltage as a function of the longitudinal magnetic field (*H*_y_). Blue and red circles correspond to data collected for the up and down magnetizations. The solid lines are parabolic and linear fit to first and second harmonic data. (d) Magnetization switching induced by pulsed electric current in SrIrO_3_/ferromagnet with different longitudinal magnetic fields at room temperature. The pulse width is 200 μs. (e) Comparison of spin Hall angle θ_SH_ or damping-like (DL)-torque efficiency *ξ_DL_* and spin Hall conductance σ_SH_ among heavy metals, TMOs and topological semimetals. Points on the dashed lines have the same ideal switching power proportional to $\frac{1}{{{\xi }_{DL}{\sigma }_{SH}}}$. (f) Comparison of switching current density *J_SW_* and power dissipation density *P*_D_ among heavy metals, TMOs and topological semimetals.

The band structure of the degenerate Dirac point produced by non-symmorphic symmetry promises to generate large Berry curvature for efficient charge–spin conversion. Additionally, the observed spin–momentum locked surface state can further contribute to charge–spin conversion by the Edelstein effect. The magnitude of SOT originating from charge–spin conversion was hence measured to evaluate its potential in spintronic devices. We performed standard second harmonic Hall measurement of SOT on SrIrO_3_ (23 nm)/Ti(3 nm)/Pt(1 nm)/Co(1.2 nm)/Pt(1 nm) heterostructures at room temperature. The SrIrO_3_ film was prepared by PLD, followed by *in situ* sputtering of a magnetic Ti/Pt/Co/Pt multilayer on top. The samples were fabricated in a Hall bar configuration for measurement. The Hall measurement obtained by scanning the perpendicular magnetic field, shown in Fig. 4b, demonstrates the perpendicular magnetic anisotropy (PMA) of the magnetic layer, which enables estimation of the SOT efficiency by harmonic Hall voltage analysis. When sweeping the transverse (*H*_y_) and longitudinal (*H*_x_) magnetic field, the first and second harmonic Hall voltage show typical linear and parabolic characteristics (Fig. 4c and d). The damping-like and field-like spin–orbit effective field (*H*_DL_ and *H*_FL_) can be obtained using Equations 1 and 2: [58]


(1) \begin{eqnarray*}
{H}_{DL\left( {FL} \right)} = - 2\frac{{{B}_{DL\left( {FL} \right)} \pm 2\eta {B}_{DL\left( {FL} \right)}}}{{1 - 4{\eta }^2}},
\end{eqnarray*}

(2) \begin{eqnarray*}
{B}_{DL\left( {FL} \right)} = {\left\{ {\frac{{\partial V_{xy}^{2\omega }}}{{\partial H}}\Big/\frac{{{\partial }^2V_{xy}^{1\omega }}}{{\partial {H}^2}}} \right\}}_{{H}_{x\left( y \right)}},
\end{eqnarray*}

where the ratio of the planar Hall voltage to the anomalous Hall voltage, *η*, is neglected due to the small planar Hall effect compared to the anomalous Hall effect in Co. By fitting the field-dependent first and second harmonic voltages, and substituting the coefficients into Equations 1 and 2, the damping-like and field-like effective fields can be determined. By further varying the applied current, as shown in Fig. S10b, the ratio between the effective field and current density in SrIrO_3_ can be obtained with *H_DL_/j_SIO_* of 388 Oe/10^11^ A/m^2^. Further, the spin Hall conductivity and SOT efficiency can be evaluated by ${\sigma }_{DL} = \frac{{2e}}{\hbar }\frac{{{\mu }_0{M}_s{t}_{Co}{H}_{DL}}}{{\mathrm{E}}}$ and ${\xi }_{DL} = \frac{{2e}}{\hbar }\frac{{{\mu }_0{M}_s{t}_{Co}{H}_{DL}}}{{{j}_{SIO}}}$ respectively, where *μ*_0_ is the vacuum magnetic permeability, t_Co_ of 1.2 nm is the thickness of the sputtered Co layer and M_s_ is the magnetization of the Co layer determined to be 1550 emu/cm^3^. Accordingly, the damping-like spin Hall conductivity *σ*_DL_ is evaluated to be 0.96 × 10^5^ $\mathrm{h}$^-^ /2e Ω^−1^m^−1^, and the damping-like SOT efficiency *ξ*_DL_ is 2.26. The exceptionally high SOT efficiency and spin Hall conductance establish this system as a highly promising candidate for manipulating the magnetic moment with minimal power consumption. A comparison of the SOT efficiencies and spin Hall conductances of many materials is listed in Table S1 and plotted in Fig. 4e. The comparison shows that the ideal energy consumption of magnetization switching in hexagonal SrIrO_3_, which is proportional to $\frac{1}{{{\xi }_{DL}{\sigma }_{DL}}}$, is lower than that reported in material systems including heavy metals, other transition metal oxides (TMOs) and topological semimetals. In particular, in comparison to the orthorhombic perovskite phase of SrIrO_3_, the hexagonal SrIrO_3_ exhibits superior performance, which manifests that lattice symmetry engineering enables the improvement of SOT efficiency.

To confirm the energy efficiency of SrIrO_3_ in switching the magnetic moment, it is important to further demonstrate magnetization switching and evaluate the energy consumption. The same sample structure for SOT measurements was utilized for magnetization switching by pulsed current with a pulse width of 200 μs. Figure 4d shows the Hall resistance *R*_xy_ as a function of the writing current density *J*_e_. With the application of an in-plane magnetic field along the current direction, deterministic switching is realized. The critical switching current density is determined to be around 5.9 × 10^5^ A/cm^2^, which leads to the power dissipation density ${P}_{\rm D} = J_c^2\rho $ of 0.8 × 10^15^ W·m^3^. A comparison of switching current density and power dissipation density, as listed in Table S1 and plotted in Fig. 4f, demonstrates that the switching current and power in hexagonal SrIrO_3_ are lower than most reported values in heavy metals, TMOs and topological materials. Therefore, the large SOT efficiency, low ideal switching energy, low switching current density and power dissipation consistently confirm the exceptional performance of hexagonal SrIrO_3_ for spintronic applications.

The giant SOT efficiency in SrIrO_3_ is realized with a large spin Hall conductivity of 0.96 × 10^5^ $\mathrm{h}$^-^ /2e Ω^−1^m^−1^ and electric conductivity of 4.2 × 10^4^ Ω^−1^m^−1^. For this conductivity, SrIrO_3_ is likely to enter a bad metal regime where the spin Hall conductivity significantly decreases with reduced conductivity similar to the observations in Pt [59] and orthorhombic SrIrO_3_ [46]. Accordingly, its spin Hall conductance and SOT performance can be further enhanced by improving its conductivity. The higher conductive state in bulk single-crystal hexagonal SrIrO_3_ [50,60] presents ample opportunity for conductivity optimization in the thin film. In addition, although the high deposition temperature is incompatible with the silicon back-end-of-line (BEOL) process, epitaxial growth on sacrificial layers (e.g. Sr_3_Al_2_O_6_) offers a viable strategy to bypass thermal constraints by transferring high-quality SrIrO_3_ film onto silicon substrates [61,62]. The high spin Hall conductivity and SOT efficiency in hexagonal SrIrO_3_ are attributed to the observed spin–momentum locked surface states and bulk band structure with degeneracies produced by non-symmorphic symmetry, which contribute to large Berry curvature. The electronic structure of the non-symmorphic Dirac point and the superior SOT performance demonstrate that engineering non-symmorphic symmetry is a promising approach to induce topological band structures and generate large SOT. Comparatively, the orthorhombic phase of SrIrO_3_ hosts Dirac nodal lines also protected by non-symmorphic symmetry [38,63–65], which similarly facilitate efficient charge–spin conversion. The presence of symmetry-protected topological features, whether Dirac nodal points in the hexagonal phase or nodal lines in the orthorhombic phase, establishes SrIrO_3_ as an ideal platform for exploring the interplay between non-symmorphic symmetry, topological electronic structure, and charge–spin conversion.

Notably, non-symmorphic symmetry is prevalent among the 230 space groups and plays a crucial role in the emergence of unique topological states and the protection of exotic fermions by enforcing band crossings in the electronic band structure of certain topological materials [37,66]. In particular, non-symmorphic symmetry protects exotic fermions, including Dirac, Möbius and hourglass fermions [31,32,35,36], each with distinct band degeneracies. Despite the wealth of theoretical predictions and experimental discoveries, the investigation of SOT effects in these non-symmorphic symmetry-protected topological states is still in its early stages. Therefore, our findings in hexagonal SrIrO_3_ establish a new material platform for systematically exploring the interplay between non-symmorphic symmetry, topological electronic states and spin transport properties. Extending this research to other non-symmorphic material systems will provide deeper insights into the fundamental role of symmetry in determining the topological electronic structure and SOT response, potentially leading to the discovery of novel TQMs with enhanced spin–charge conversion efficiency and opening up new avenues for spintronic device applications.

## CONCLUSION

In summary, our study reveals that hexagonal SrIrO_3_ is a symmetry-enforced topological Dirac semimetal exhibiting unprecedentedly giant SOT, directly visualizing its pronounced spin–momentum locked surface state and band degeneracy through comprehensive ARPES and non-linear transport measurements. These unique electronic features drive remarkably efficient charge–spin conversion, resulting in record-high damping-like torque and low-current magnetization switching. Our findings establish non-symmorphic symmetry engineering as a cornerstone strategy for realizing next-generation spintronic materials. Beyond advancing the fundamental understanding of the interplay between non-symmorphic symmetry, topology and SOT, this work initiates a paradigm shift in materials design for energy-efficient spintronics, ultimately paving the way for revolutionary applications in ultra-fast, energy-efficient magnetic memory and logic devices. Further exploration of non-symmorphic topological materials promises to unlock their full potential to revolutionize future information technologies.

## METHODS

### Sample preparation and structural characterization

The hexagonal SrIrO_3_ films were grown on SrTiO_3_(111) substrates by PLD with a krypton fluoride (KrF) (λ = 248 nm) excimer laser and high reflection high-energy electron diffraction (RHEED). During the deposition of the SrIrO_3_ films, the substrate’s temperature, oxygen partial pressure and laser fluence were set to 700°C, 0.1 mbar and 1.5 J/cm^2^ at a frequency of 2 Hz, respectively. For second harmonic Hall measurement of SOT, the Ti/Pt/Co/Pt multilayer was *in situ* deposited on SrIrO_3_ by magnetron sputtering in the chamber connected to the PLD chamber. The film structure was characterized using XRD with four-circle goniometry (D8 Discover, 106 Bruker AXS) with Cu *K*${\alpha }_{1,2}$ radiation on a diffractometer equipped with a Ge(002) crystal monochromator.

### ARPES

*In situ* ARPES measurements were performed at the Surface and Interface Spectroscopy beamline of the Swiss Light Source. The beamline is equipped with a PLD setup directly connected to the ARPES end-station, ensuring that samples remain in an ultrahigh vacuum environment prior to measurements. Data were acquired at a temperature of ~15 K, with photon energies varying from 40 to 126 eV. The energy and momentum resolutions were approximately 20 meV and 0.2°, respectively.

### Device fabrication and electrical measurements

The SrIrO_3_ films and heterostructures were patterned into a Hall bar by photolithography and Ar^+^ ion etching. The harmonic Hall and resistance measurements were performed using a Keithley 6221 current source and two SR830 lock-in amplifiers. The low-temperature measurements with the magnetic field were performed in an Oxford cooling system with a superconducting magnet. For the magnetization switching measurement, a pulsed current with a duration of 200 ${\mathrm{\mu s}}$ was applied, and a DC current of 0.1 mA was applied for reading the Hall resistance 2 s after the current pulse.

### Computational methods

The structural and electronic properties of the hexagonal SrIrO_3_ were computed using the Vienna *Ab initio* Simulation Package (VASP) [67] with the projector augmented-wave method [68] and Perdew–Burke–Ernzerhof revised for solids (PBEsol) [69] scheme. The kinetic energy cutoff was set to 500 eV, and the Brillouin zone was sampled with a 9 × 9 × 3 Γ-centered grid. The relaxed lattice constants were *a* = *b* = 5.736, *c* = 14.343 Å. Spin–orbit coupling (SOC) was also self-consistently included. By using the VASP2WANNIER interface [70], Ir-5*d* and O-2*p* orbitals were projected in order to construct the tight binding model. The Fermi surface at the Γ(0,0,0) and A(0,0,0.5) planes was computed using WANNIERTOOLS [71]. Irreducible representations were calculated with Irvsp software [72].

## Supplementary Material

nwag077_Supplemental_File
