# Supplementary material for: Symmetry-enforced topological Dirac semimetal for giant spin–orbit torque with ultralow power dissipation
Source: Natl Sci Rev. 2026 Feb 4;13(7):nwag077. doi: 10.1093/nsr/nwag077 (PMC13105180; doi:10.1093/nsr/nwag077)
Supplement: nwag077_Supplemental_File [file nwag077_supplemental_file.docx]

**Supplementary Materials**

**Giant spin-orbit torque in a symmetry-enforced topological Dirac semimetal**

**Table of Contents**

**Supplemental Note 1. Influence of substrate symmetry on the crystal structure of SrIrO_3_ films.**

**Supplemental Note 2. Topological Dirac semimetal protected by non-symmorphic symmetry.**

**Supplemental Note 3. Calculation of spin Hall conductivity**

**Supplemental Note 4. Discussion on the surface state and bulk state in ARPES measurement.**

**Supplemental Note 5. Experimental evidence of Dirac points in ARPES measurements.**

**Supplemental Note 6. Temperature dependent nonlinear planar Hall effect.**

**Supplemental Note 7. Evaluation of fieldlike torque and current dependent SOT measurement.**

**Supplemental Note 8. Thermal effect in second harmonic Hall measurement of SOT.**

**Supplemental Note 9. Shunting factor in SrIrO_3_/Ti/Pt/Co/Pt.**

**Supplemental Note 10. Discussion on magnetization switching and power dissipation.**

**Supplemental Fig. S1: Crystal structure of SrIrO_3_..**

**Supplemental Fig. S2: DFT calculated structure of hexagonal SrIrO_3_.**

**Supplemental Fig. S3: Supplemental Figure S3: DFT calculated structure of hexagonal SrIrO_3_ in slab.**

**Supplemental Fig. S4: DFT calculated surface state of hexagonal SrIrO_3_.**

**Supplemental Fig. S5: DFT calculated spin Hall conductivity and berry curvature.**

**Supplemental Fig. S6: Calculated band dispersions.**

**Supplemental Fig. S7: ARPES measurements of band dispersion in *k*_x_-*k*_y_ plane.**

**Supplemental Fig. S8:** **ARPES measurements of band dispersion in *k*_x_-*k*_z_ plane.**

**Supplemental Fig. S9: Nonlinear planar Hall measurements.**

**Supplemental Fig. S10: Second harmonic Hall voltage measurements with low magnetic field.**

**Supplemental Fig. S11: Second harmonic Hall voltage measurements with large magnetic field.**

**Supplemental Fig. S12: Comparison of SOT efficiencies between SIO/Ti/Pt/Co/Pt and the control sample (Ti/Pt/Co/Pt).**

**Supplemental Table S1: Comparison of spin current generating layers and parameters in switching perpendicular magnetic layer.**

**Supplemental Note 1. Influence of substrate symmetry on the crystal structure of SrIrO_3_ films.**

The crystal structure of SrIrO_3_ films is strongly influenced by the symmetry and orientation of the substrate on which it is grown. As reported in our previous publication [1] and demonstrated in Fig. S1, SrIrO_3_ adopts an orthorhombic structure when grown on a SrTiO_3_(001) substrate, while it exhibits a hexagonal structure when grown on a SrTiO_3_(111) substrate (Fig. S1(c,d)). The crystal structure of hexagonal SrIrO_3_ with face-shared oxygen octahedral can accommodate three different symmetries belonging to different space groups, including the monoclinic C2/c (no. 15), orthorhombic Cmcm (no. 63) and trigonal P$\overline{3}$1c (no. 163). The differences in symmetry among these structures arise from various oxygen octahedral distortions, as shown in Fig. S2 (a). In the bulk state, the monoclinic C2/c (no. 15) structure exhibits the lowest energy (Fig. S2 (b)). However, when SrIrO_3_ is grown on the SrTiO_3_(111) substrate, the C3 symmetry of the substrate can significantly influence the energy landscape of the different crystal structures. The highly distorted Ir-O-Ti bonds at the interface between SrIrO_3_ with C2/c _­_(no.15) space group results in an increased energy, making it comparable to that of SrIrO_3_ with Cmcm (no. 63) and P$\overline{3}$1c (no. 163) space groups (Fig. S3). The C3 symmetry of the SrTiO_3_(111) substrate favors the formation of SrIrO_3_ structures with compatible symmetry, such as the trigonal P$\overline{3}$1c (no. 163) space group. Notably, after structural relaxation, the relaxed SrIrO_3_ structure tends to be similar to the trigonal P$\overline{3}$1c (no. 163) space group (Fig. S3 (a)), further supporting the influence of the substrate's C3 symmetry on the crystal structure of SrIrO_3_. Consequently, SrIrO_3_ grown on SrTiO_3_(111) likely adopts a hexagonal structure with the trigonal P$\overline{3}$1c space group, due to the symmetry constrains imposed by the substrate.

**Supplemental Note 2. Topological Dirac semimetal protected by non-symmorphic symmetry.**

We consider three possible space groups for the hexagonal SrIrO_3_ films constrained by the SrTiO_3_(111) substrate: C2/c (no. 15), Cmcm (no. 63), and P$\overline{3}$1c (no. 163). Despite these differences, all these structures possess non-symmorphic symmetry elements, such as glide planes and screw axes, which plays a crucial role in protecting the Dirac points. The shared non-symmorphic symmetry in these structures is the glide symmetry, denoted as $\mathbf{G}=\{M|(0,0,0.5)\}$. This symmetry operation consists of a reflection denoted as **M** with a plane parallel c axis, followed by a half translation along the c axis. Due to the glide symmetry of the lattice, the Bloch state in the **M**-invariant lines is simultaneously an eigenstate of the glide operator **G** and Hamiltonian. Applying the glide operation twice generates a translation along c axis, i.e. $\mathbf{G}^{2}=-e^{-i\mathbf{k}\cdot\mathbf{c}}$, where the minus sign is due to the spin of electrons. Hence, for Bloch state in the **M**-invariant lines we have

$\boldsymbol{G}|\left. u\left( \boldsymbol{k} \right) \right\rangle=\pm ie^{-i\boldsymbol{k}\cdot c/2}|\left. u\left( \boldsymbol{k} \right) \right\rangle$, (S1)

which leads to $\boldsymbol{G}|\left. u\left( \mathbf{k}+\mathbf{K} \right) \right\rangle=\pm ie^{-i(\mathbf{k+K)}\cdot\mathbf{c}/2}|\left. u\left( \mathbf{k}+\mathbf{K} \right) \right\rangle$, where $\mathbf{K}$ is an odd reciprocal lattice vector along the c axis. For **K** being the odd reciprocal lattice vector along c axis, $e^{i\mathbf{K}\cdot\mathbf{c}/2}=-1$, thus

$\boldsymbol{G}|\left. u\left( \boldsymbol{k}+\boldsymbol{K} \right) \right\rangle=\mp ie^{-\boldsymbol{ik}\cdot\boldsymbol{c}/2}|\left. u\left( \boldsymbol{k}+\boldsymbol{K} \right) \right\rangle$, (S2)

Equation (2) shows that the two eigenstate of **G** swap their eigenvalues when traversing an odd reciprocal lattice vector along the c axis. As a result, the two bands associated with these eigenstates will intersect an odd number of times as they cross the Brillouin zone (BZ). For SrIrO_3_ film preserving time-reversal symmetry T and inversion symmetry P, Kramers degeneracy exists for all k states, meaning that each electronic state is doubly degenerate. Additionally, the energy dispersion is an even function of k, i.e. E(-k)=E(k). Hence, for Dirac point with four fold degeneracy at **k**=**K**/2 (i.e. L and A points in BZ), the crossing of two bands with different eigenvalues of **G** across the BZ is maintained, and the even function of energy dispersion is preserved. This demonstrates that the Dirac points are protected by the non-symmorphic symmetry.

**Supplemental Note 3. Calculation of spin Hall conductance**

We projected the Bloch wavefunction into Wannier function and constructed tight binding model Hamiltonian. By using the tight binding model Hamiltonian the spin Hall conductivity was calculated in the Kubo formula approaching in linear response.

The calculated spin Hall conductivity shown in Fig. S5 reaches magnitude of ${10}^{4} \frac{\hbar}{2e}\Omega^{-1}m^{-1}$ which is comparable to the measured value.

**Supplemental Note 4. Discussion on the surface state and bulk state in ARPES measurement.**

As shown in Fig. 2(b), the ARPES measurement with varied photon energy reveals 2D surface state. The difference of intensity map at different photon energies, such as 71 eV and 103 eV, still indicates the observation of 3D bulk states. By comparison to the DFT calculation result of bulk state Fermi surface map demonstrated in Fig. 2(e)(f) and surface state Fermi surface map in Fig. S4 (b), we can identify the measurement at 71 eV contains signal from the A plane of bulk state while the signals at 103 eV correspond to the $\Gamma$ plane of bulk state. The measured band dispersion at 71 eV shown in Fig. S4 (a) and 103 eV shown in Fig. 2(g) both exhibits a hole pocket at H, L or M, K which matches the calculated surface state in Fig. S4. The band dispersion at A or $\Gamma$ varies for measurement at 71 eV and 103 eV, with a much weaker signal at $\Gamma$ for 71 eV. We attribute this observation to a mixing of surface state and bulk state at A or $\Gamma$. The calculated surface state exhibits signals of an electron pocket at $\bar{\Gamma}$ as shown in Fig. S4, while the calculated 3D band structure only exhibit signals at A as shown in Fig. 2(g). Thus, the observed band structure at 71 eV mainly contains the signal of surface states while the band structure measured at 103 eV contains both signals of surface states and bulk states. The comparison of ARPES results and DFT calculation in hence distinguishes the surface state and bulk state.

**Supplemental Note 5. Experimental evidence of Dirac points in ARPES measurements.**

Dirac points are theoretically predicted at the boundary of the Brillouin zone in hexagonal SrIrO_3_ due to its non-symmorphic symmetry. According to DFT calculations shown in Fig. 1e, there are two Dirac points near the Fermi level: one at the L point and another at the A point of the BZ. ARPES measurements help to visualize the possible existence of the Dirac point at the A point. Fig. S8 (a) and (b) show the second derivative of energy dispersion along the L-A-L and H-A-H directions, respectively, exhibiting linear characteristics around the A point. The stacking plot of constant energy maps in the k_x_-k_y_ plane (Fig. S8 (c)) also shows symmetric linear energy dispersion along different directions in the k_x_-k_y_ plane. To further investigate the energy dispersion along the k_z_ direction, ARPES measurements were performed with varied photon energies. The stacking plot of constant energy maps in the k_x_-k_z_ plane (Fig. S8(d)) displays similar energy dispersion, confirming the 3D nature of the Dirac point at A point of the BZ. These ARPES results provide strong evidence for the existence of the theoretically predicted 3D Dirac points in hexagonal SrIrO_3_.

**Supplemental Note 6. Temperature dependent nonlinear planar Hall effect.**

Nonlinear planar Hall effect is measured at different temperatures. The temperature dependent NPHE signal is shown in Fig. S9(a). At low temperature near 10K, the NPHE signal exhibits a peak around 20K. This behavior together with the heating effect can explain the deviation from linear relation between nonlinear planar Hall resistance and applied current in Fig. 3f in the main text. When increasing temperature, the NPHE signal reduces and undergoes a sign change around 250K. The NPHE signal at room temperature shown in Fig. S9(b) exhibits opposite sign in comparison to that measured at low temperatures. This sign change of NPHE signal may indicate different mechanisms of NPHE with different carrier lifetime dependence [2] , which can be further explored.

**Supplemental Note 7. Evaluation of fieldlike torque and current dependent SOT measurement.**

The fieldlike torque is measured by collecting first and second harmonic Hall voltage when sweeping the transverse magnetic field as shown in Fig. S10(a). The fieldlike torque effective field can be evaluated by equations (1) and (2) in the main text. The Oestered field is subtracted according to Ampere's law (H_oersted_=I/2W). By varying the applied current and calculate the effective field as plotted in Fig. S10(b). Then the fieldlike spin Hall conductivity and fieldlike torque efficiency can be evaluated by $\sigma_{DL,FL}=\frac{2e}{\hbar}\frac{\mu_{0}M_{s}t_{Co}H_{FL}}{E}$ and $\xi_{FL}=\frac{2e}{\hbar}\frac{\mu_{0}M_{s}t_{Co}H_{FL}}{j_{SIO}}$. The fieldlike spin conductivity is 1.82$\times{10}^{\boldsymbol{5}}\hbar$**/**2e $\Omega^{-1}m^{-1}$ and fieldlike torque efficiency is about 0.42.

**Supplemental Note 8. Thermal effect in second harmonic Hall measurement of SOT.**

In the second harmonic Hall measurement, the thermoelectric effect will be included and may affect the evaluation of the SOT effective field. The thermal effect can arise from temperature gradient in the device in three directions, i.e., $\nabla_{x}T, \nabla_{y}T, \nabla_{z}T$. The second harmonic Hall voltage from thermal effect can originate from Seebeck effect, spin Seebeck effect(SSE) [3], anomalous Nernst effect (ANE) and Nernst effect. For the in-plane magnetic field, the Nernst effect doesn’t contribute. The Seebeck effect will generate a constant voltage when sweeping magnetic fields and it will not affect the slope of linear relations of second harmonic Hall voltages, thus will not influence the evaluation of SOT. ANE and SSE are thus the only possible thermal effect that can affect the evaluation of SOT.

For the ANE and SSE, as the temperature gradient and thermoelectric current should be perpendicular to ANE generated Hall current or inverse spin Hall current , the $\nabla_{y}T$ which is along the Hall measurement direction will not generate the ANE or SSE affecting the measurement. Temperature gradients along the other two directions can generate ANE and SSE proportional to $\nabla_{x}TM_{z}$ and $\nabla_{z}TM_{x}$. For ANE and SSE proportional to $\nabla_{x}TM_{z}$, when sweeping the in-plane magnetic field, the thermal voltage is even for positive and negative field. In the second harmonic Hall voltage as a function of in-plane magnetic field shown in Fig. S10(b) and Fig. 4 (c)(d), the good linear relation between second harmonic Hall voltage and magnetic field, which is an odd function, manifests the negligible contributions from the term of $\nabla_{x}TM_{z}$.

Therefore, the only thermal term that can affect the evaluation of SOT is proportional to $\nabla_{z}TM_{x}$, the magnitude of which can be estimated as follows. When sweeping the longitudinal magnetic field larger than H_K_ to align the magnetization in-plane, the second harmonic Hall resistance can be expressed by  [4]

$R_{xy}^{2\omega}=\frac{R_{A}}{2}\frac{H_{DL}}{{|H}_{x}|-H_{k}}+R_{ANE+SSE}\frac{H_{x}}{{|H}_{x}|}+R_{offset}$ (S3)

where the term proportional to planar Hall effect and field like effective field is neglected. By fitting second harmonic signal in positive and negative large magnetic field range, the thermal signal arising from ANE and SSE can be obtained. As shown in Fig. S10(a), the difference of second harmonic resistance at large field limit between positive and negative magnetic field corresponds to thermal signal generated by vertical temperature gradient proportional to $\nabla_{z}TM_{x}$. Here magnetization is totally aligned in x direction at large field, so M_x_ is just the total magnetization. At small magnetic field, the thermal resistance proportional to $\nabla_{z}TM_{x}=\nabla_{z}T\sqrt{M^{2}-{(M_{z})}^{2}}$ can be evaluated by determining M_z_ through the first harmonic signal. In this way, the thermal effect when sweeping small longitudinal magnetic field can be estimated, which is shown in Fig. S10 (c). In comparison to the measured second harmonic signal as shown in Fig. S10(b), the thermal signal is about two orders smaller, which can in hence be neglected.

**Supplemental Note 9. Shunting factor in SrIrO_3_/Ti/Pt/Co/Pt.**

In the measurement of SOT efficiency and magnetization switching, the current density is calculated by considering the current only flowing through SrIrO_3_ layer, which requires determination of shunting current through magnetic layer. We separately measured the resistivity of SrIrO_3_ grown on SrTiO_3_(111) of 2354 $\mu\Omega\cdot cm$ and the resistivity of Ti/Pt/Co/Pt layer of 135$\mu\Omega\cdot cm$. Then using the parallel resistance model and considering the thicknesses of the film, the shunting factor defined as s=I_FM_/I_SIO_ is determined to be 4.7 using ${\rho_{SIO}t_{FM}}/{\rho_{FM}t_{SIO}}$.

**Supplemental Note 10. Discussion on SOT efficiency and energy efficiency in switching magnetization.**

For applications in SOT devices, it’s important to evaluate the energy consumption of switching magnetization. There are two ways to estimate the power dissipated during magnetization switching. The first approach is to utilize the measured SOT efficiency and spin Hall conductivity to calculate the ideal switching current and corresponding energy consumption. As the switching current is proportional to $\frac{1}{\xi_{DL}}$, the switching power is proportional to $\frac{1}{\xi_{DL}^{2}\sigma}=\frac{1}{\xi_{DL}\sigma_{DL}}$, where $\xi_{DL}$ is SOT efficiency, $\sigma$ is charge conductivity and $\sigma_{DL}$ is spin Hall conductivity. Comparison of $\xi_{DL}$ and $\sigma_{DL}$ among typical heavy metals, transition metal oxides and topological materials are listed in Table S1 and plotted in Fig. 4(e). As shown in Fig. 4(e), the large $\xi_{DL}$ and $\sigma_{DL}$ makes the ideal switching power smaller than most material systems.

The second approach to evaluate switching energy is using the critical switching current density in the experiment of magnetization switching to calculate the power dissipation density defined as $P_{D}=J_{c}^{2}\rho$. There are various factors can affect the observed critical switching current density J_c_. As switching perpendicular and in-plane magnetization can differ greatly in switching current, we will discuss and comparing the result for switching perpendicular magnetization. Using the simplified model in macrospin limit, J_c_ can be expressed by  [5]

$J_{c}=\frac{e\mu_{0}M_{s}t(H_{K}-\sqrt{2}|H_{x}|)}{\hbar\xi_{DL}}$. (S4)

The model considering the domain wall pinning yields [6]

$J_{c}=\frac{4e\mu_{0}M_{s}tH_{c}}{\pi\hbar\xi_{DL}}$. (S5)

In both equation (S4) and (S5), *e* is the electron charge; $\hbar$ is the reduced Planck constant; $\mu_{0}$ is the vacuum permeability; *H_x_* is the magnetic field along current direction; *t*, *M_s_*, *H_k_*, *H_c_* are thickness, magnetization, anisotropy field and coercive field of magnetic layer. Further, for current pulse width of 0.2 ms we used and that reported in most researches, the switching enters thermal activation regime, where switching current density *J_sw_* is expressed by [7]

$J_{sw}=J_{c}(1-\frac{k_{b}T}{E}ln\frac{\tau_{p}}{\tau_{0}})$, (S6)

where E is the energy barrier, $\frac{1}{\tau_{0}}$ is the attempt frequency associated with the precession frequency of magnetization with typical value of 10 GHz. Accordingly, the current pulse width affecting the energy barrier, temperature during switching can have impact on the observed critical switching current. Therefore, the factors influencing the critical switching current include the coercive field *H_c_*, anisotropy field *H_k_*, magnetization *M_s_*, thickness of magnetic layer, SOT efficiency $\xi_{DL}$, in-plane magnetic field *H_x_* and current pulse width $\tau_{p}$. Table S1 makes comparison of critical switching current and dissipation power density among typical heavy metal, topological insulator and topological semimetals in switching perpendicular magnetization. The comparison of switching current density and power dissipation density is plotted in Fig. 4(f). The switching current density J_sw_ is comparable to that in topological insulator and much smaller than other heavy metals and topological semimetals. The power dissipation density P_D_ is comparable to the best values in topological insulator and topological semimetals. As the parameters in our experiment of H-SIO/Ti/Pt/Co/Pt including *H_c_* of 150Oe, *H_k_* of 2100 Oe, M_s_ of, *H_x_* of 200 Oe, $\tau_{p}$ of 0.2 ms are comparable to that reported in other researches, the observed energy efficient switching of perpendicular magnetization is reliable. Therefore, both approaches yield high performance of hexagonal SrIrO_3_ in switching magnetization.


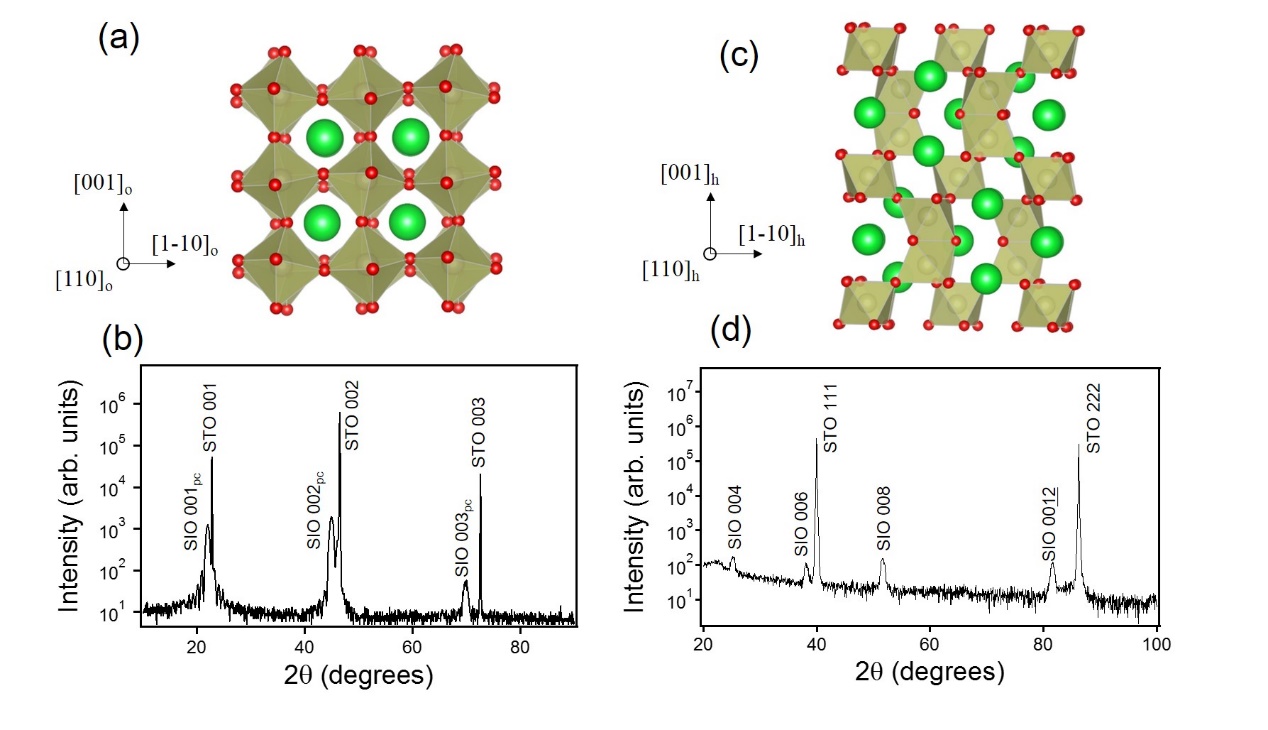


**Supplemental Fig. S1: Crystal structure of SrIrO_3_.** (a) Lattice structure of orthorhombic SrIrO_3_. (b) XRD $\theta$-2$\theta$ scan of orthorhombic SrIrO_3_ film grown on SrTiO_3_ (001) substrate. (c) Lattice structure of hexagonal SrIrO_3_. (d) XRD $\theta$-2$\theta$ scan of hexagonal SrIrO_3_ film grown on SrTiO_3_ (111) substrate.


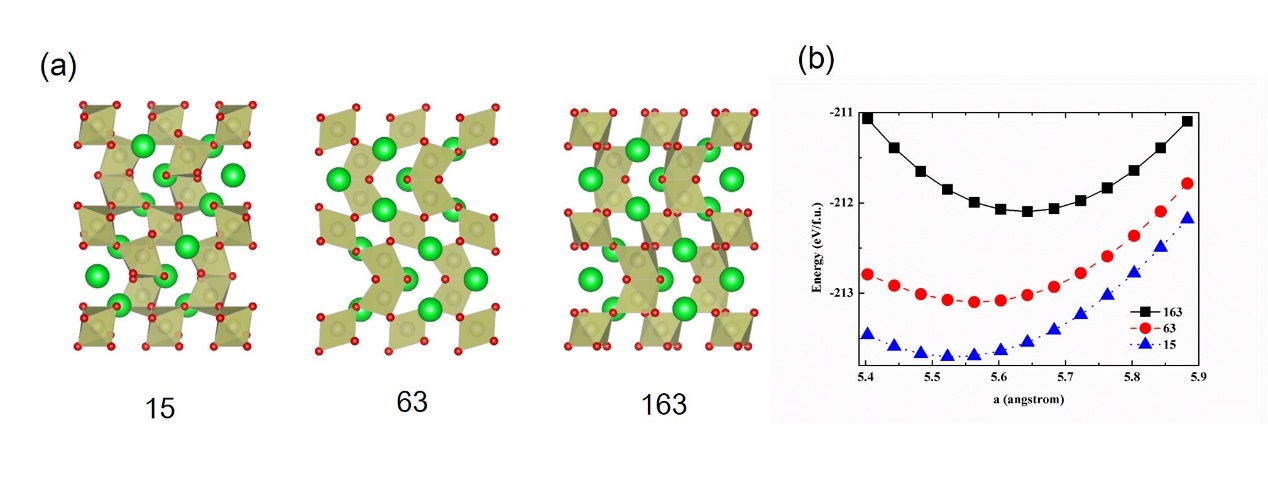


**Supplemental Fig. S2: DFT calculated structure of bulk hexagonal SrIrO_3_.** (a) Three possible lattice structures of hexagonal SrIrO_3_ belonging to 15, 63 and 163 space groups. (b) The formation energy of SrIrO_3_ with different lattice symmetry.


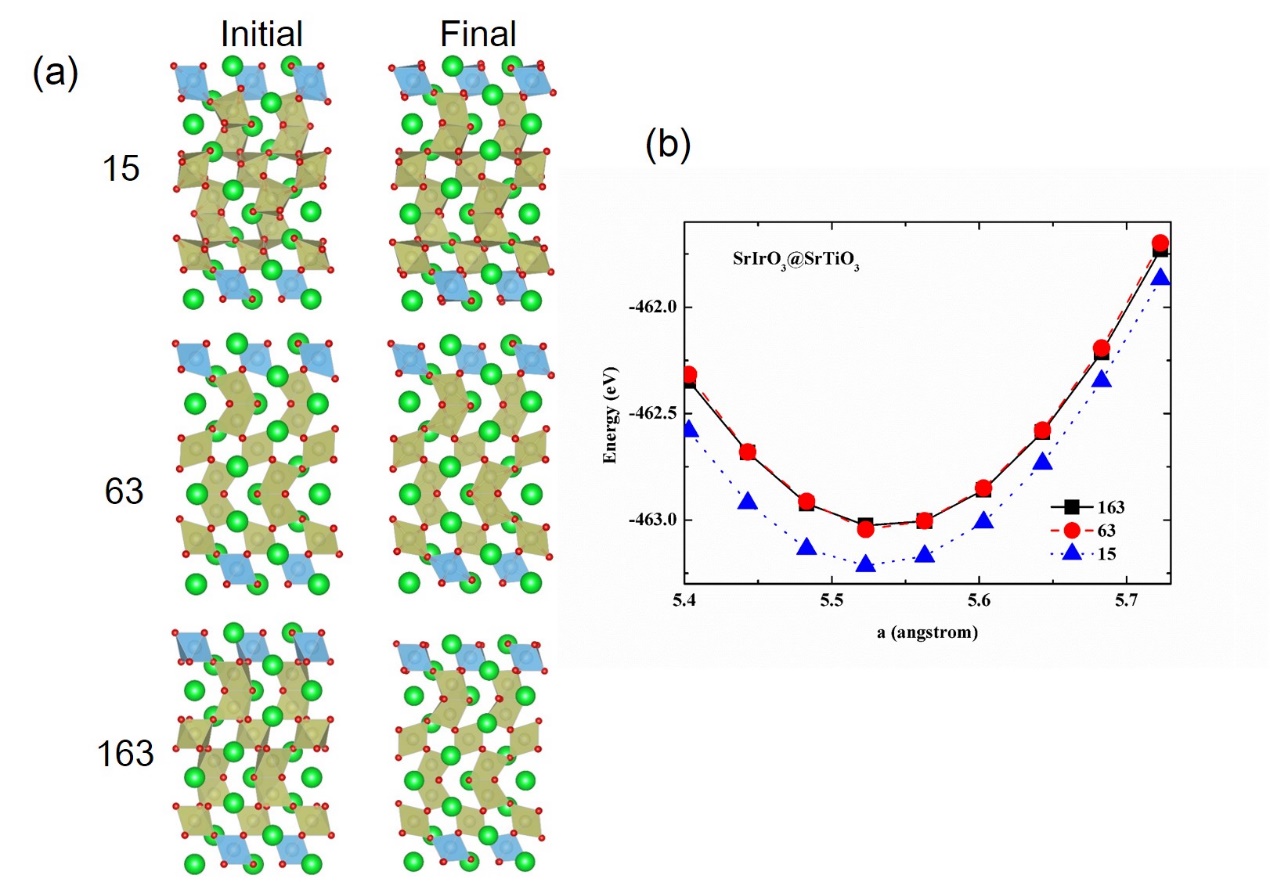


**Supplemental Fig. S3: DFT calculated structure of hexagonal SrIrO_3_ in slab.** (a) Three possible lattice structures of hexagonal SrIrO_3_ constrained by the SrTiO_3_(111). The left column shows the initial structure and the right column displays the structure after structure relaxation. (b) The formation energy of SrIrO_3_ with different lattice symmetry after relaxation.

**Supplemental Fig. S4: DFT calculated surface state of hexagonal SrIrO_3_.** (a) DFT calculations of energy dispersion of surface state. (b) Fermi surface map of surface state.

**Supplemental Fig. S5: DFT calculated spin Hall conductivity and berry curvature.** (a) Spin Hall conductivity as a function of Fermi energy (b) Distribution of Berry curvature in the BZ.

**Supplemental Fig. S6: Calculated band dispersions.** Band dispersions along H-A-H, L-A-L and H-L-H high symmetric directions near Fermi level.

**Supplemental Fig. S7: ARPES measurements of band dispersion in *k*_x_-*k*_y_ plane.** Energy dispersion (upper row) and its second derivative (lower row) measured at photon energy of 71eV corresponding to the $\Gamma$ plane of the Brillouin zone.


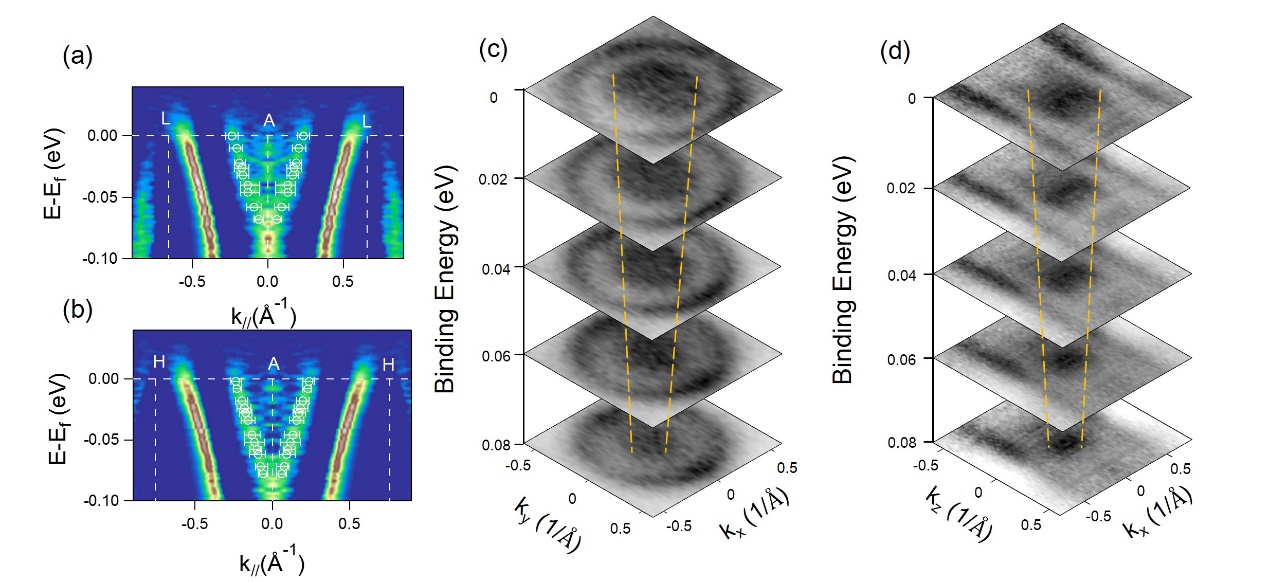


**Supplemental Fig. S8: ARPES measurements of band dispersion in *k*_x_-*k*_z_ plane.** (a) (b) Second derivative of energy dispersion along L-A-L and H-A-H respectively measured at photon energy of 103 eV. Peak fits to momentum distribution curves (MDC) are shown as the symbols.(c) (d) Stacking plot of isoenergy map along k_x_-k_y_ and k_x_-k_z_ at different binding energies shows band structure of a Dirac cone.


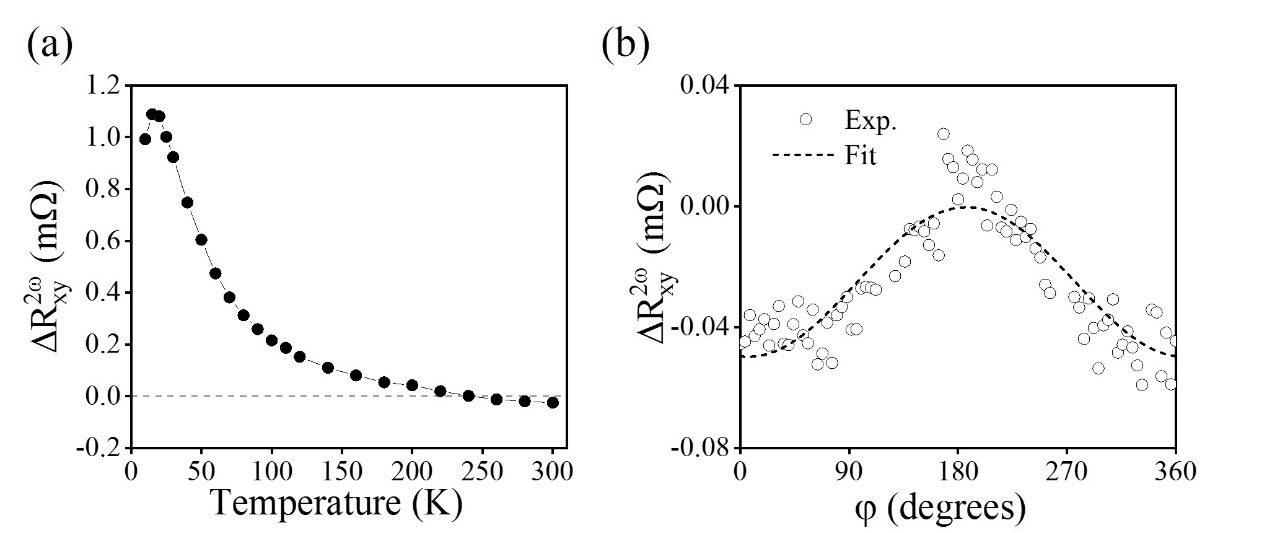


**Supplemental Fig. S9: Nonlinear planar Hall measurements.** (a) The amplitude of nonlinear planar Hall signal as a function of temperature measured at magnetic field of 7 T and current of 0.8 mA. (b) Nonlinear planar Hall effect at room temperature measured with magnetic field of 7 T and current of 0.8 mA.


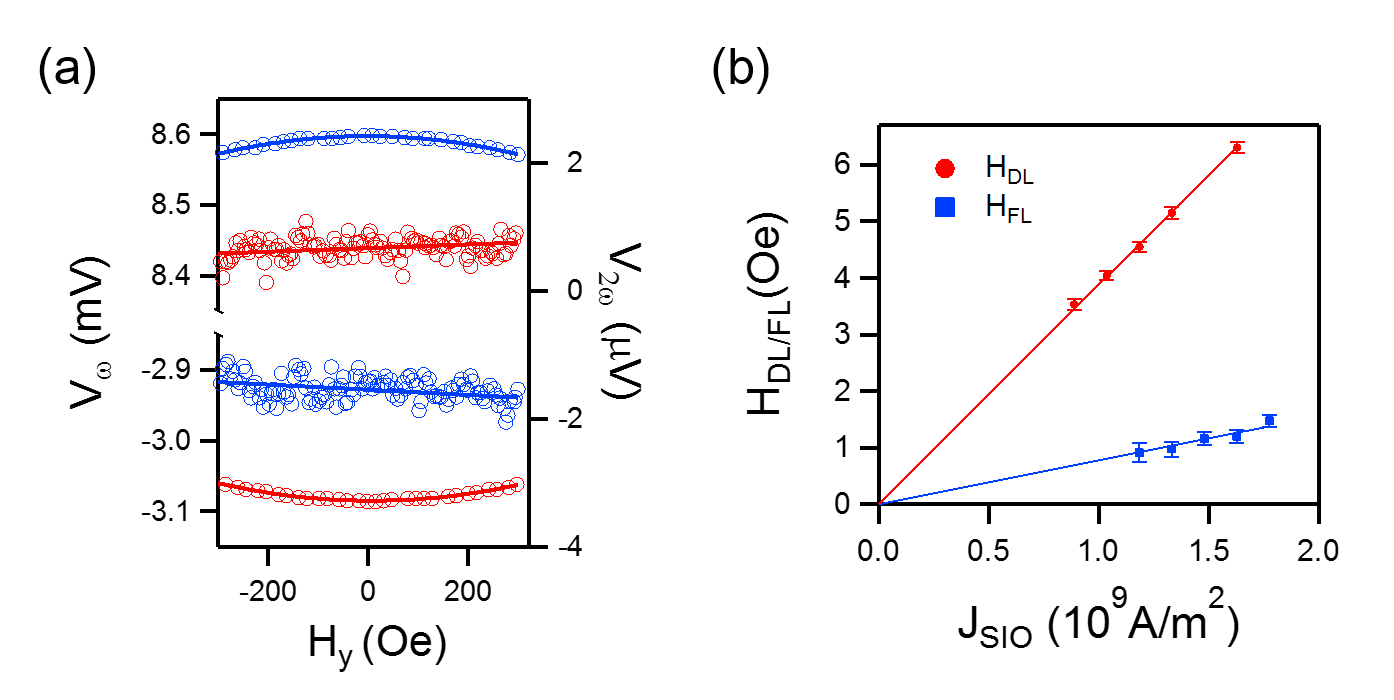


**Supplemental Fig. S10: Second harmonic Hall voltage measurements with low magnetic field.** (a) Typical first and second harmonic voltage when sweeping transverse magnetic field (H_y_) measured with current of 1.8 mA. Blue and red circles correspond to data collected for the up and down magnetizations. The solid lines are parabolic and linear fit to first and second harmonic data.(b) Calculated dampinglike (H_DL_) and fieldlike (H_FL_) effective field as a function of applied current density in SIO.


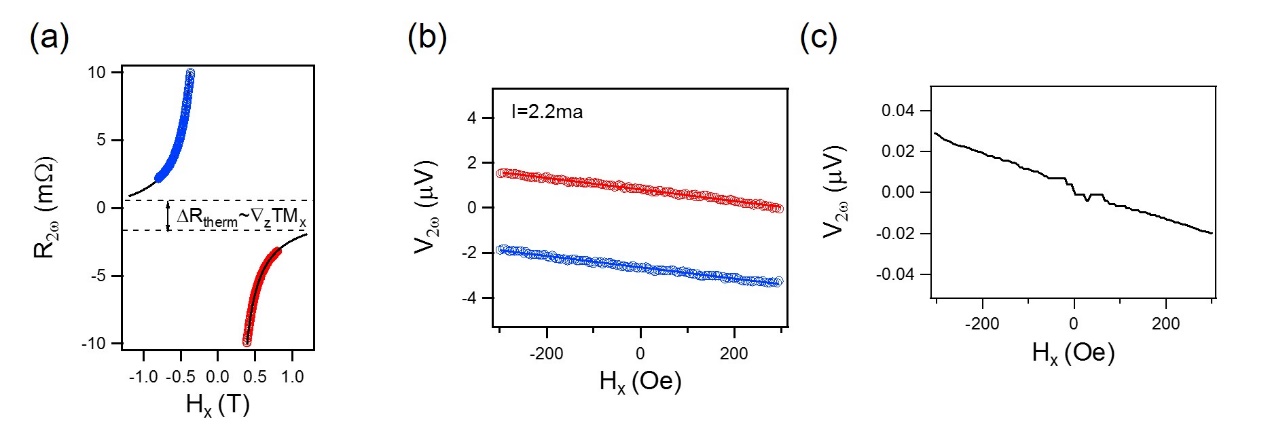


**Supplemental Fig. S11: Second harmonic Hall voltage measurements with large magnetic field.** (a) Second harmonic Hall resistance as a function of longitudinal magnetic field at large field range with fitting curves measured 2.2 mA. (b) Second harmonic Hall voltage as a function of longitudinal magnetic field at small field range. (c) Estimated thermal contribution to second harmonic Hall voltage as a function of small longitudinal field.


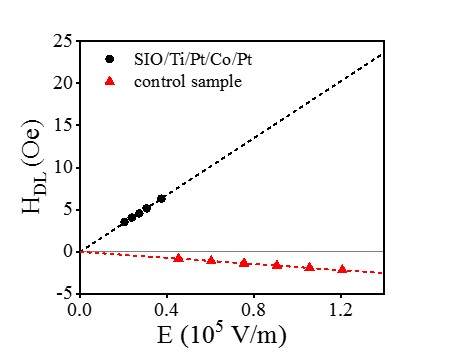


**Supplemental Fig. S12: Comparison of SOT efficiencies between SIO/Ti/Pt/Co/Pt and the control sample (Ti/Pt/Co/Pt).** Dampinglike effective field characterized by second harmonic Hall measurement as a function of electric field in SIO/Ti/Pt/Co/Pt and the control sample (Ti/Pt/Co/Pt).

| Materials | $\boldsymbol{\xi}_{\boldsymbol{DL}}$ | $\boldsymbol{\sigma}_{\boldsymbol{DL}}$($\mathbf{10}^{\boldsymbol{5}}$  $\boldsymbol{\hbar}$/2e $\boldsymbol{\Omega}^{\boldsymbol{-1}}\boldsymbol{m}^{\boldsymbol{-1}}$) | | $\boldsymbol{\sigma(}\mathbf{10}^{\boldsymbol{5}}$  ${\boldsymbol{\Omega}^{\boldsymbol{-1}}\boldsymbol{m}}^{\boldsymbol{-1}}\mathbf{)}$ | | Switching  Magnetic layer | $\boldsymbol{J}_{\boldsymbol{sw}}\boldsymbol{(}\boldsymbol{10}^{\boldsymbol{6}}$  $\boldsymbol{A\cdot}\boldsymbol{cm}^{\boldsymbol{-2}}\boldsymbol{)}$ | $\boldsymbol{P}_{\boldsymbol{D}}\boldsymbol{(}\boldsymbol{10}^{\boldsymbol{15}}$  $\boldsymbol{W\cdot}\boldsymbol{m}^{\boldsymbol{3}}\boldsymbol{)}$ | | | H_k_  (kOe) | H_c_  (Oe) | | H_x_  (Oe) | Ref. | |  |
| --- | --- | --- | --- | --- | --- | --- | --- | --- | --- | --- | --- | --- | --- | --- | --- | --- | --- |
| H-SIO | 2.26 | 0.96 | 0.42 | | Pt/Co/Pt | | 0.59 | | 0.8 | 2.1 | | 150 | 200 | | | This work | |
| SrIrO_3_ | 0.1 | 0.16 | 1.6 | | Fe_(1-x)_Ga_x_ | | 3 | | 5.6 | 3 | | 100 | 600 | | | [8] | |
| SrRuO_3_ | 0.04 | 0.25 | 6.3 | | Fe_(1-x)_Ga_x_ | | 4.5 | | 3.2 | 2.5 | | 50 | 200 | | | [8] | |
| SrRuO_3_ | 0.21 | 0.47 | 2.2 | | CoPt | | 3.8 | | 6.5 | - | | 100 | 200 | | | [9] | |
| Pt(MgO) | 0.28 | 3.8 | 13.5 | | Co/MgO | | 11.5 | | 9.8 | 20 | | 80 | 1500 | | | [10] | |
| Pt | 0.16 | 4.9 | 30 | | Co/MgO | | 18.4 | | 11.3 | 20 | | 80 | 1500 | | | [10] | |
| Pt | 0.08 | 3.4 | 42.5 | | Co/AlO_x_ | | 28.5 | | 19.1 | 2.8 | | 150 | 100 | | | [11] | |
| $\boldsymbol{\beta}$-Ta | 0.15 | 0.78 | 5.2 | | CoFeB/MgO | | 7.8 | | 11.7 | - | | 70 | 100 | | | [12] | |
| (BiSb)_2_Te_3_ | 2.5 | 0.46 | 0.18 | | CoFeB/MgO | | 0.52 | | 1.5 | 2.2 | | 30 | 100 | | | [13] | |
| Bi_0.9_Sb_0.1_ | 52 | 130 | 2.5 | | MnGa | | 1.5 | | 0.9 | 40 | | 5000 | 3500 | | | [14] | |
| Bi_x_Se_(1-x)_ | 8.67 | 0.68 | 0.078 | | CoFeB/Ga/CoFeB | | 0.43 | | 1.29 | - | | 30 | 80 | | | [15] | |
| Bi_2_Se_3_ | 1.75 | 0.43 | 0.243 | | Py(IMA) | | 0.6 | | 1.48 | - | | - | - | | | [16] | |
| TaIrTe_4_(a) | 0.05 | 0.246 | 4.9 | | CoFeB/MgO | | 2.35 | | 1.13 | - | | 10 | 0 | | | [17] | |
| TaIrTe_4_(a) | 0.113 | 0.54 | 4.8 | | CoFeB/MgO | | 7.652 | | 11.7 | 5 | | 40 | 0 | | | [18] | |
| TaIrTe_4_(b) | 0.28 | 0.3 | 1.1 | | CoFeB/MgO | | 1.02 | | 0.95 | - | | 10 | 30 | | | [17] | |
| WTe_2_(a) | 0.15 | 0.12 | 0.8 | | CoFeB/MgO | | 2.25 | | 5.7 | 5 | | 40 | 0 | | | [19] | |
| WTe_2_(a) | 0.013 | 0.032 | 2.4 | | CoFeB/MgO | | 1.3 | | 0.7 | - | | 10 | 300 | | | [17] | |
| WTe_2_(b) | 0.026 | 0.038 | 1.4 | | CoFeB/MgO | | 1.09 | | 0.85 | - | | 10 | 300 | | | [17] | |
| WTe_2_ | 0.2 | 0.1 | 0.53 | | CoTb | | 0.7 | | 0.92 | - | | 100 | 900 | | | [20] | |
| PtTe_2_ | 0.05-0.15 | 0.2-1.6 | 3-30 | | CoTb | | 9.9 | | 3.32 | - | | 200 | 200 | | | [21] | |
| Cd_3_As_2_ | 0.1 | 0.13 | 1.3 | | - | | - | | - | - | | - | - | | | [22] | |

**Supplemental Table S1: Comparison of spin current generating layers and parameters in switching perpendicular magnetic layer**.

**References**

[1] X. Zheng, S. Kong, J. Zhu, J. Feng, Z. Lu, H. Du, and B. Ge, Layer-by-Layer Epitaxial Growth of Monoclinic SrIrO_3_ Thin Films on (111) - Oriented SrTiO_3_ through Interface Engineering, Thin Solid Films **709**, 138119 (2020).

[2] J. Y. Ba, Y. M. Wang, H. J. Duan, M. X. Deng, and R. Q. Wang, Nonlinear Planar Hall Effect Induced by Interband Transitions: Application to Surface States of Topological Insulators, Phys. Rev. B **108**, 241104 (2023).

[3] M. Schreier, N. Roschewsky, E. Dobler, S. Meyer, H. Huebl, R. Gross, and S. T. B. Goennenwein, Current Heating Induced Spin Seebeck Effect, Appl. Phys. Lett. **103**, 242404 (2013).

[4] M. Hayashi, J. Kim, M. Yamanouchi, and H. Ohno, Quantitative Characterization of the Spin-Orbit Torque Using Harmonic Hall Voltage Measurements, Phys. Rev. B **89**, 144425 (2014).

[5] K. S. Lee, S. W. Lee, B. C. Min, and K. J. Lee, Threshold Current for Switching of a Perpendicular Magnetic Layer Induced by Spin Hall Effect, Appl. Phys. Lett. **102**, 112410 (2013).

[6] T. Y. Chen, C. Te Wu, H. W. Yen, and C. F. Pai, Tunable Spin-Orbit Torque in Cu-Ta Binary Alloy Heterostructures, Phys. Rev. B **96**, 104434 (2017).

[7] R. H. Koch, J. A. Katine, and J. Z. Sun, Time-Resolved Reversal of Spin-Transfer Switching in a Nanomagnet, Phys. Rev. Lett. **92**, 088302 (2004).

[8] A. Tang et al., Implementing Complex Oxides for Efficient Room-Temperature Spin–Orbit Torque Switching, Adv. Electron. Mater. **2200514**, 1 (2022).

[9] S. Li, B. Lao, Z. Lu, X. Zheng, K. Zhao, L. Gong, T. Tang, K. Wu, R. W. Li, and Z. Wang, Room Temperature Spin-Orbit Torque Efficiency and Magnetization Switching in SrRuO3 -Based Heterostructures, Phys. Rev. Mater. **7**, 24418 (2023).

[10] L. L. Zhu, L. L. Zhu, M. Sui, D. C. Ralph, and R. A. Buhrman, Variation of the Giant Intrinsic Spin Hall Conductivity of Pt with Carrier Lifetime, Sci. Adv. **5**, 1 (2019).

[11] L. Liu, O. J. Lee, T. J. Gudmundsen, D. C. Ralph, and R. A. Buhrman, Current-Induced Switching of Perpendicularly Magnetized Magnetic Layers Using Spin Torque from the Spin Hall Effect, Phys. Rev. Lett. **109**, 1 (2012).

[12] L. Liu, C. F. Pai, Y. Li, H. W. Tseng, D. C. Ralph, and R. A. Buhrman, Spin-Torque Switching with the Giant Spin Hall Effect of Tantalum, Science **336**, 555 (2012).

[13] H. Wu et al., Room-Temperature Spin-Orbit Torque from Topological Surface States, Phys. Rev. Lett. **123**, 207205 (2019).

[14] N. H. D. Khang, Y. Ueda, and P. N. Hai, A Conductive Topological Insulator with Large Spin Hall Effect for Ultralow Power Spin–Orbit Torque Switching, Nat. Mater. **17**, 808 (2018).

[15] M. Dc et al., Room-Temperature High Spin–Orbit Torque Due to Quantum Confinement in Sputtered BixSe(1–x) Films, Nat. Mater. **17**, 800 (2018).

[16] Y. Wang et al., Room Temperature Magnetization Switching in Topological Insulator-Ferromagnet Heterostructures by Spin-Orbit Torques, Nat. Commun. **8**, 6 (2017).

[17] Y. Zhang et al., Room Temperature Field-Free Switching of Perpendicular Magnetization through Spin-Orbit Torque Originating from Low-Symmetry Type II Weyl Semimetal, Sci. Adv. **9**, eadg9819 (2023).

[18] Y. Liu et al., Field-Free Switching of Perpendicular Magnetization at Room Temperature Using out-of-Plane Spins from TaIrTe4, Nat. Electron. **6**, 732 (2023).

[19] F. Wang et al., Field-Free Switching of Perpendicular Magnetization by Two-Dimensional PtTe_2_/WTe_2_ van Der Waals Heterostructures with High Spin Hall Conductivity, Nat. Mater. **23**, 768 (2024).

[20] C. W. Peng, W. B. Liao, T. Y. Chen, and C. F. Pai, Efficient Spin-Orbit Torque Generation in Semiconducting WTe2with Hopping Transport, ACS Appl. Mater. Interfaces **13**, 15950 (2021).

[21] H. Xu et al., High Spin Hall Conductivity in Large-Area Type-II Dirac Semimetal PtTe_2_, Adv. Mater. **32**, 2000513 (2020).

[22] W. Yanez et al., Spin and Charge Interconversion in Dirac-Semimetal Thin Films, Phys. Rev. Appl. **16**, 054031 (2021).
